# The Protein Kinase *SmSnRK2.6* Positively Regulates Phenolic Acid Biosynthesis in *Salvia miltiorrhiza* by Interacting with *SmAREB1*

**DOI:** 10.3389/fpls.2017.01384

**Published:** 2017-08-09

**Authors:** Yanyan Jia, Zhenqing Bai, Tianlin Pei, Kai Ding, Zongsuo Liang, Yuehua Gong

**Affiliations:** ^1^College of Life Sciences, Northwest A&F University Yangling, China; ^2^College of Life Sciences, Zhejiang Sci-Tech University Hangzhou, China; ^3^Sichuan Tea College, Yibin University Yibin, China

**Keywords:** *Salvia miltiorrhiza*, SnRK2 protein, AREB/ABFs, ABA, overexpression, phenolic acids

## Abstract

Subclass III members of the sucrose non-fermenting-1-related protein kinase 2 (SnRK2) play essential roles in both the abscisic acid signaling and abiotic stress responses of plants by phosphorylating the downstream ABA-responsive element (ABRE)-binding proteins (AREB/ABFs). This comprehensive study investigated the function of new candidate genes, namely *SmSnRK2.3*, *SmSnRK2.6*, and *SmAREB1*, with a view to breeding novel varieties of *Salvia miltiorrhiza* with improved stress tolerance stresses and more content of bioactive ingredients. Exogenous ABA strongly induced the expression of these genes. PlantCARE predicted several hormones and stress response *cis*-elements in their promoters. *SmSnRK2.6* and *SmAREB1* showed the highest expression levels in the leaves of *S. miltiorrhiza* seedlings, while *SmSnRK2.3* exhibited a steady expression in their roots, stems, and leaves. A subcellular localization assay revealed that both *SmSnRK2.3* and *SmSnRK2.6* were located in the cell membrane, cytoplasm, and nucleus, whereas *SmAREB1* was exclusive to the nucleus. Overexpressing *SmSnRK2.3* did not significantly promote the accumulation of rosmarinic acid (RA) and salvianolic acid B (Sal B) in the transgenic *S. miltiorrhiza* hairy roots. However, overexpressing *SmSnRK2.6* and *SmAREB1* increased the contents of RA and Sal B, and regulated the expression levels of structural genes participating in the phenolic acid-branched and side-branched pathways, including *SmPAL1*, *SmC4H*, *Sm4CL1*, *SmTAT*, *SmHPPR*, *SmRAS*, *SmCHS*, *SmCCR*, *SmCOMT*, and *SmHPPD*. Furthermore, SmSnRK2.3 and SmSnRK2.6 interacted physically with SmAREB1. In summary, our results indicate that *SmSnRK2.6* is involved in stress responses and can regulate structural gene transcripts to promote greater metabolic flux to the phenolic acid-branched pathway, via its interaction with *SmAREB1*, a transcription factor. In this way, *SmSnRK2.6* contributes to the positive regulation of phenolic acids in *S. miltiorrhiza* hairy roots.

## Introduction

*Salvia miltiorrhiza* Bunge (family Labiatae) is one of the traditional bulk medicinal materials. Its dried roots have been prescribed for the clinical treatment of many human diseases, such as irregular menstruation, cardiovascular and cerebrovascular diseases, and inflammation (Kai et al., 2011; Ma et al., 2013). This plant contains two main bioactive ingredients: lipid-soluble tanshinones and water-soluble phenolic acids (Chen et al., 2001). Phenolic acids have attracted considerable attention, largely due to their important medicinal effects and their convenient extraction by decoction, the main mode of application in traditional Chinese medicine. At present, the biosynthetic pathway of phenolic acids and its competition bypass branches in *S. miltiorrhiza* have been proposed, and the key enzyme genes involved in the phenolic acid-branched and side-branched pathways have been cloned (Xiao et al., 2011; Ma et al., 2013; Zhang Y. et al., 2014).

Faced with an ever-increasing demand for *S. miltiorrhiza,* its supply from wild resources is no longer sufficient. In response, many studies have since focused on increasing this species phenolic acid contents by either overexpressing or suppressing its transcription factors or key enzyme genes within the relevant metabolic pathways. For example, the overexpression of *SmTAT*, *SmC4H*, *SmHPPR*, *AtPAP1*, *AtEDT1*, and *SmPAP1* induced a substantial accumulation of phenolic acids in the transgenic *S. miltiorrhiza* (Zhang Y. et al., 2010; Xiao et al., 2011; Hao et al., 2016; Liu Y. et al., 2016), whereas the downregulation of *SmMYB39*, *SmHPPD*, *SmCCR1*, or *SmCHS* all increased the content of phenolic acids (Xiao et al., 2011; Wang et al., 2012; Zhang et al., 2013; Zhang et al., 2015).

However, the effective components of *S. miltiorrhiza* are in the form of secondary metabolites. Because their quality and quantity strongly depends on environmental stresses, such as drought, cold, and salinity, this results in a lower yield of *S. miltiorrhiza* (Akula, 2011; Liu et al., 2011; Zhao et al., 2014). Several studies have reported that by overexpressing *SmLEA2*, *AtDREB1A*, *AtDREB1B*, and *AtDREB1C*, the salt and drought tolerance of transgenic *S. miltiorrhiza* was improved (Wei et al., 2016a,b, 2017; Wang et al., 2017). More of such genes should be investigated via genetic engineering methods, as it could assist in breeding new varieties of *S. miltiorrhiza* that have a higher content of active ingredients and a stronger tolerance to stresses.

Sucrose non-fermenting 1 (SNF1)-related protein kinase 2 (SnRK2) is a plant-specific Serine/Threonine (Ser/Thr) protein kinase family. Based on their amino acid sequence similarity, the members of the SnRK2 family have been grouped into three subclasses. Compared to the subclass I/II members, those of subclass III play central roles in the positive regulation of abscisic acid (ABA) signaling and in the responses to osmotic stress (Fujii and Zhu, 2009). Overexpression of subclass III SnRK2 subfamily members *SAPK9*, *TaSnRK2.8*, and *ZmSAPK8* could enhance drought, salt, or cold tolerance in transgenic plants. The subclass III SnRK2 reportedly regulate the expression of the ABA-responsive gene, primarily through four ABA-responsive element (ABRE)-binding proteins (AREB/ABFs): ABF1, ABF2/AREB1, ABF3, and ABF4/AREB2, all of which are highly inducible by osmotic stress and ABA treatments applied to vegetative tissues (Yoshida et al., 2010, 2015; Fujita et al., 2013).

Although the SnRK2 family has received extensive and in-depth research, their subclass III members in *S. miltiorrhiza* have not yet been explored. Moreover, previous studies in our laboratory showed that exogenous ABA, as an elicitor, is capable of promoting the accumulation of phenolic acids (Cui et al., 2012). Therefore, it is possible that the subclass III SnRK2 members from *S. miltiorrhiza* have key roles to play in the synthesis of phenolic acids in this plant. With this in mind, we aimed to identify the orthologous genes of SnRK2s in *S. miltiorrhiza*, and to then evaluate whether these proteins contribute to the regulation of phenolic acid accumulation and whether they are involved in the stress responses of this plant. In this study, we not only cloned and functionally characterized the two subclass III SnRK2 members (i.e., *SmSnRK2.3* and *SmSnRK2.6*), but we also identified and functionally characterized *SmAREB1*, which has a close affinity with *AtAREB1*. Additionally, we analyzed the physical interaction between SmSnRK2.3/2.6 and SmAREB1. These results not only fill a gap in our knowledge of the subclass III SnRK2 genes in *S. miltiorrhiza*, but they also provide a theoretical foundation to improve the stress tolerance and bioactive ingredient contents of *S. miltiorrhiza* via genetic engineering. More broadly, this study further contributes to the preliminary exposition of the molecular mechanism underpinning the exogenous ABA-induced accumulation of phenolic acid content in plants.

## Materials and Methods

### Plant Material and Treatments

Mature *S. miltiorrhiza* seeds were collected from the Dan-shen cultivation base of the Shaanxi Tasly plant medicine Co. Ltd. (Shangluo, China). These seeds were utilized to obtain sterile plantlets as previously reported (Yan and Wang, 2007). Sterilized seedlings were cultured on an MS medium (pH 5.8) that contained 7% agarose. The hairy roots of *S. miltiorrhiza* were derived from these plantlets, infected with *Agrobacterium rhizogenes* (ATCC15834 strain), and sub-cultured every 30 days.

The seeds were sown in plugs and hydroponically grown at 25°C, under continuous light, for 2 months. Fresh roots, stems, and leaves were separately harvested from the ensuing seedlings, which were used as samples to determine expression levels of *SmSnRK2.3/2.6* and *SmAREB1* in different tissues of *S. miltiorrhiza*.

For the ABA treatment, the ensuing seedlings were sprayed with 100 μM ABA solution. The samples were harvested at 0, 0.5, 1, 3, 6, 9, 12, 24, and 48 h after treatment. All of these collected samples were immediately frozen in liquid nitrogen and stored at -80°C prior to analysis.

### Total RNA and DNA Extraction

Total complete RNA was isolated from the frozen *S. miltiorrhiza* samples by using the RNAprep Pure Plant Kit (TIANGEN, China). The RNA was then reversely transcribed to generate the cDNA, according to manufacturer’s instructions of the PrimeScript^TM^ RT reagent Kit with gDNA Eraser (Takara, Japan). To isolate the genomic DNA, an improved cetyltrimethylammonium bromide (CTAB) method was used. The quality and concentration of the genomic DNA and RNA were determined by using 0.8% agarose gel electrophoresis and a nucleic acid spectrometer (NanoDrop ND-1000, Thermo Scientific).

### Gene Isolation and Bioinformatics Analysis

Based on a previously established *S. miltiorrhiza* transcriptome database (Shao et al., 2016), local BLAST analyses were performed using three subclass III SnRK2 members of *Arabidopsis thaliana* and an *SmAREB1* fragment, which was amplified by using degenerate primers, as queries. According to the obtained sequences, we designed three pairs of specific primers to perform the PCR amplification with the *pfu* DNA polymerase (Thermo, United States). Amplification templates were genomic DNA and cDNA reversely transcribed via RNA, both of which were extracted from the *S. miltiorrhiza* hairy roots. The procedure for the PCR reaction system followed the manufacturer’s instructions. The PCR products were gel purified by a DNA Gel Extraction Kit (OMEGA, United States) and then cloned into the pEASY-Blunt Simple cloning vector (TransGen Biotech, China) for sequencing. The primer sequences are shown in Supplementary Table S1.

Both the ORF-finder^1^ and the GENSCAN Web Server^2^ were used to confirm the open reading frame (ORF) of each gene. Amino acid sequences of the genes were deduced with the ProtParam tool^3^ and submitted to the NCBI database^4^ for BLASTP searches. The DNAMAN software was used to perform the multiple alignments. Sequence alignment results were combined with an online motif scan tool^5^ to analyze the functional domain of proteins. Phylogenetic trees were generated with the MEGA7 software program, by employing the neighbor-joining method, with 1000 bootstrap replicates. All amino acid sequences of the other species were downloaded from the NCBI database^6^. The intron and exon distributions of the genes were analyzed by the online GSDS2.0 software^7^ (Hu et al., 2015).

### Isolation and Analysis of the Promoters

Based on the sequences identified from the *S. miltiorrhiza* genome (Xu et al., 2016), the gene-specific primers were designed to cover the 2,048 bp 5′ flanking sequence of *SmSnRK2.3*, 1,865 bp of *SmSnRK2.6*, and 1,911 bp of *SmAREB1*. The *cis*-acting elements of the promoter regions were predicted by using PlantCARE^8^ (Lescot et al., 2002). The primers used to amplify the promoters are listed in Supplementary Table S2.

### Quantitative Real-Time PCR Analysis

Total RNA was extracted from the *S. miltiorrhiza* hairy roots or *S. miltiorrhiza* seedlings, according to the method described above. The RNA samples (1 μg) were then reverse transcribed to 20 μL cDNA using the PrimeScript^TM^ RT reagent Kit with gDNA Eraser (Takara, Japan), according to manufacturer’s instructions. The resulting cDNA was diluted to 150 ng/μL with DNase/RNase-Free H_2_O. Quantitative real-time PCR (qRT-PCR) was performed in the CFX96 Real-Time PCR System (Bio-Rad, United States). The 20 μL reaction mixture contained 10 μL of 2 × SYBR^®^ Premix Ex Taq^TM^ II (Perfect Real Time, Takara, Japan), 1.6 μL of a forward/reverse primer (10 μM), 1.6 μL of a cDNA template, and 15.2 μL of DNase/RNase-Free H_2_O. The PCR procedure went as follows: at 95°C for 30 s, then 40 cycles of 95°C for 5 s and 60°C for 30 s, this program was followed by a melting curve analysis (65–95°C with temperature increment of 0.5°C every 5 s). In this study, the qRT-PCR data for the genes responding to the ABA treatment were normalized to β*-actin* (Yang et al., 2012; Wei et al., 2017), while the other qRT-PCR data were normalized to β*-actin* and *ubiquitin* (Yang et al., 2010; Xiao et al., 2011). All the primers used for the qRT-PCR analysis are listed in Supplementary Table S3.

### Subcellular Localization Analysis

The modified-vector pCAMBIA1301 containing *eGFP* was used in this study. The coding regions of the *SmSnRK2.3*, *SmSnRK2.6*, and *SmAREB1* genes (without a stop codon) were successfully amplified and cloned upstream of the *eGFP* gene in the *BamH*I-cleaved pCAMBIA1301 binary vector, to generate the GFP fusion vectors *SmSnRK2.3-*p1301, *SmSnRK2.6-*p1301, and *SmAREB1-*p1301. Subsequently, the *SmAREB1-*p1301 construct and the pCAMBIA1301 empty vector were transformed into onion epidermal cells via gene gun, and the transformed onion cells were later examined under an A1 confocal microscope (Nikon, Japan). To locate nuclei, the tissues were incubated with 4, 6-diamidino-2-phenylindole dihydrochloride (DAPI). A transient expression assay in *Nicotiana benthamiana* leaves was performed to determine the subcellular localization of *SmSnRK2.3* and *SmSnRK2.6.* The *Agrobacterium tumefaciens* (EHA105 strain) suspension cultures, harboring either the construct *SmSnRK2.3-*p1301 or *SmSnRK2.6-*p1301, were, respectively, infiltrated into leaves of *N. benthamiana* along with a plasma membrane marker, following the method described by Zhang H. et al. (2014). The plasma membrane marker was based on a full-length fusion of the aquaporin *PIP2A* to red fluorescent proteins (Nelson et al., 2007). To serve as the control, the *A. tumefaciens* (EHA105 strain) suspension cultures containing the empty vector pCAMBIA1301 were infiltrated into the leaves of *N. benthamiana*. After 3–4 days, the *N. benthamiana* leaves were harvested and subjected to a chimeric fluorescence signal analysis under an A1 confocal microscope (Nikon, Japan). The primers used for the subcellular localization analysis are listed in Supplementary Table S4.

### Analysis of *SmAREB1* Transcriptional Activity

The transactivation activity of *SmAREB1* was examined by the yeast one-hybrid assay, a method widely used to measure the transactivation ability of transcription factors (Shukla et al., 2006; Hu et al., 2013; Shen et al., 2014). The complete coding sequence of *SmAREB1* was amplified with specific primers (Supplementary Table S5). The PCR product was cloned into *EcoR*I and *BamH*I sites in the pGBKT7 vector, which contains the *Trp* reporter gene and the DNA binding domain of the transcription factor *GAL4*, to generate a *GAL4* DNA-BD-*SmAREB1* fusion plasmid, named *SmAREB1-*BD. The *AtMYB15*-pGBKT7 plasmid was constructed as a positive control. These two recombinant plasmids and the negative control pGBKT7 plasmid were used, respectively, to transform the *Saccharomyces cerevisiae* AH109 strain which carriers the *His3*, *Ade2*, and *LacZ* reporter genes under the *GAL4* promoter, following the manufacturer’s protocol (Clontech, United States). The ensuing transformants were verified via a yeast colony PCR and then plated onto synthetic dropout (SD)/-Trp and SD/-Trp-His-Ade plates cultured at 30°C for 3 days. The transcriptional activation activity was evaluated according to their growth status.

### Yeast Two-Hybrid (Y2H) Assays

The coding sequence of *SmSnRK2.3/2.6* was cloned into the pGADT7 vector, enzyme digested by *EcoR*I and *BamH*I, thus producing the construct *SmSnRK2.3/2.6*-pGADT7 (*SmSnRK2.3/2.6*-AD). The primer sequences, including the cleavage site used for the amplification, are listed in Supplementary Table S5. The plasmid combinations of *SmSnRK2.3/2.6*-AD and *SmAREB1*-BD, *SmSnRK2.3/2.6*-AD and pGBKT7, pGADT7 and *SmAREB1*-BD, as well as that of pGBKT7 and pGADT7, were co-transformed into AH109, following the manufacturer’s protocol (Clontech, United States). The AH109 cells carrying the *SmSnRK2.3/2.6*-AD + pGBKT7, pGADT7 + *SmAREB1*-BD, or pGBKT7 + pGADT7 plasmids were used as negative controls. The transformed colonies were shaken in an YPDA liquid medium until the OD_600_ of their cell density was approximately 0.6, then, they were serially diluted to fractions of 1/10, 1/100, and 1/1000 by using sterilized double-distilled water. From these yeast cell dilutions, 2 μL were taken and spotted onto SD/-Trp-Leu and SD/-Leu-Trp-His-Ade plates and incubated at 30°C for 3–5 days. Next, to test for possible interactions, the X-β-Gal staining assay was carried out following the methodology of Zhang H. et al. (2014).

### Bimolecular Fluorescence Complementation (BiFc) Analysis

To confirm the protein interactions *in* *vivo,* two plant expression binary vectors, pSPYNE-35S and pSPYCE-35S, were used in BiFc analysis (Walter et al., 2004). On the basis of the *SmSnRK2.3/2.6*-p1301 and *SmAREB1*-p1301 constructs, the pSPYCE-35S and pSPYNE-35S vectors were used to replace the pCAMBIA1301 vector at the *BamH*I restriction enzyme site to generate the recombinant plasmids *SmSnRK2.3/2.6*-pSPYNE and *SmAREB1*-pSPYCE. For the transient expression assay, these plasmids and an empty vector (negative control) were co-transformed into the *A. tumefaciens* strain EHA105, in combination with the p19 strain to infiltrate 6- week-old *N. benthamiana* leaves, as done by Zhang H. et al. (2014). The chimeric fluorescence signals of the expressed fusion proteins were detected 3–5 days after infiltration under a Fluoview FV1000 confocal microscope (Olympus, Japan).

### Purification of the *SmSnRK2.3/2.6*-GST Fusion Protein in *Escherichia coli*

The coding sequence of *SmSnRK2.3/2.6* was cloned into the pGEX-6p-1 vector, it harbored a glutathione *S*-transferase (GST) tag, by using specific primers (Supplementary Table S6). The *Escherichia coli* cells (Rosetta strain) harboring *SmSnRK2.3/2.6-*GST and the empty vector pGEX-6p-1 were induced by adding 0.5 mM isopropy-β-*D*-thiogalactoside (IPTG) for 16 h at 22°C. The *E. coli* cells were harvested via centrifugation, resuspended in pre-cooled PBS buffer, and then broken with the ultrasonic method. The soluble GST fusion proteins were purified using the ProteinIso GST Resin (TransGen Biotech, China). Then, these purified proteins were verified through the Western blotting technique.

### Identification of Phosphorylation Sites via Liquid Chromatography Tandem MS (LC-MS/MS)

To identify the phosphorylation sites of the SmSnRK2.3/2.6 protein, we separated the purified protein samples via sodium dodecyl sulfate polyacrylamide gel electrophoresis (SDS-PAGE). Specifically, the targeted protein was excised from the Coomassie Brilliant Blue (CBB) staining gel, and then it was digested via filter-aided sample preparation (FASP), as previously described (Wisniewski et al., 2009). The peptide mixtures from the gel slice were injected onto a Zorbax 300 SB-C18 peptide trap (Agilent Technologies, United States), to desalt them in an auto-sampler, and then they were separated by reverse phase capillary high performance liquid chromatography (HPLC), using a RP-C18 column (0.15 mm × 150 mm, Column Technology Inc., United States). Prior to this, the chromatographic column had been balanced using a 95% solution A, and the mobile phases were solution A (0.1% formic acid in water) and solution B (0.1% formic acid and 84% acetonitrile in water). Samples were eluted with the following linear gradient: solution B increased from 4 to 50% in the first 30 min, then increased to 100% in the next 4 min, after which it was kept constant at 100% for 1 min.

After separation and desalination via HPLC, samples were analyzed by tandem mass spectrometry, performed on a Q Exactive mass spectrometer (Thermo Fisher, United States) equipped with an electrospray interface and operated in the positive ion mode. The mass spectrometer used one full MS scan, followed by 10 MS/MS scans on the 10 most intense ions from the MS spectrum, under the following settings: repeat count of 2; repeat duration of 30 s; and an exclusion duration of 90 s. Raw MS/MS spectra were subjected to the MASCOT engine (Matrix Science, United Kingdom) against the sequence of the SmSnRK2.6 protein. For protein identification, the following parameters were used: Enzyme = Trypsin, Missed cleavage = 2, Peptide mass tolerance = 20 ppm, MS/MS tolerance = 0.1 Da, Mascot score ≥ 20, the ESI ion trap was selected for the instrument type, Carbamidomethyl (C) was the fixed modification, while Oxidation (M), Phospho (ST), and Phospho(Y) were used as the variable modifications.

### Construction of Plant Expression Vectors and Acquisition of Positive Transgenic Hairy Roots

The coding regions of *SmSnRK2.3*, *SmSnRK2.6*, and *SmAREB1* were amplified and cloned into the restriction site *spe*I of the pCAMBIA1304 binary vector, under the control of the CaMV35S promoter and the NOS terminator, respectively. The recombinant plasmids *SmSnRK2.3*-1304, *SmSnRK2.6*-1304, and *SmAREB1*-1304 were transformed into *A. rhizogenes* ATCC15834, by using the empty pCAMBIA1304 vector as a vector-only control. The transformation of leaf explants from the sterile plantlets of *S. miltiorrhiza*, which followed a previously described method (Kai et al., 2011), acquired the hairy root lines; when these reached a certain biomass, their genomic DNA was extracted for PCR identification and to screen for the positive transgenic lines. These latter lines were used for RNA and phenolic acid extraction and they were regularly sub-cultured (every 30 days). All of the primers used for the overexpression vector construction and in the PCR identification of transgenic lines are listed in Supplementary Table S7.

### HPLC Analysis of Phenolic Acid Contents

Rosmarinic acid (RA) and salvianolic acid B (Sal B) are the main phenolic acids in *S. miltiorrhiza*. while Sal B has been designated as a marker component of *S. miltiorrhiza* in the official Chinese Pharmacopoeia, RA is the synthetic precursor material (Di et al., 2013). The contents of Sal B and RA in the transgenic lines and empty vector controls (EVs) were verified by an HPLC analysis, according to the general method in our laboratory (Xing et al., 2015; Liu L. et al., 2016). The only minor difference is that a 20 mg sample of powder was dissolved in 4 mL of 70% methanol.

### Statistical Analysis

In this study, irrespective of the HPLC analysis of phenolic acid contents, or the qRT-PCR analysis of gene expression levels, all the experiments were performed in triplicate. The results are presented as means ± standard deviation (SD). Gene relative expression levels were calculated using the delta-delta Ct (2^-ΔΔCt^) method (Livak and Schmittgen, 2001; Wang et al., 2015). All data were analyzed using Statistical Package for Social Science (SPSS v.16.0) software. Significant difference (*p* < 0.05) of mean values was compared using Tukey’s multiple comparison test or student’s *t*-test (indicated in the Figure legends).

## Results

### Isolation and Bioinformatics Analysis of *S. miltiorrhiza* Genes

**FIGURE 1:**
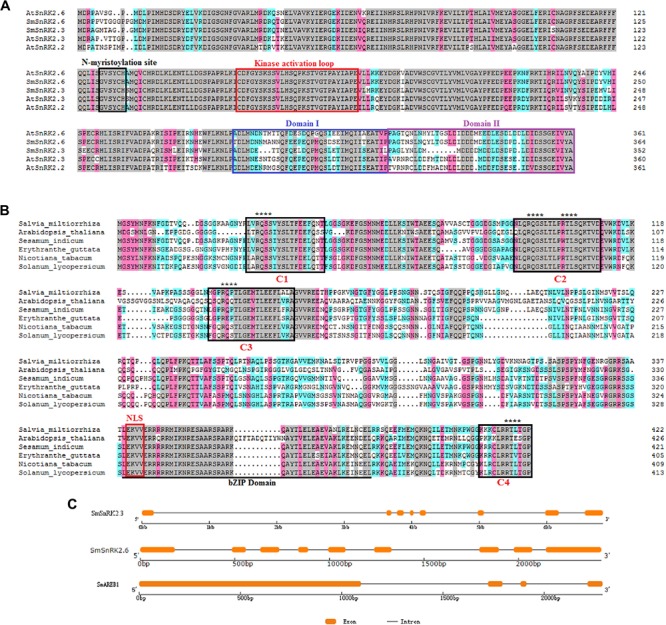
Sequence analysis of *SmSnRK2.3*, *SmSnRK2.6*, and *SmAREB1.* **(A)** Multiple alignments of the deduced amino acid sequences of SmSnRK2.3 and SmSnRK2.6 with AtSnRK2.2, AtSnRK2.3, and AtSnRK2.6 from *Arabidopsis thaliana* (At). The regions of the *N*-myristoylation site, kinase activation loop, domain I, and domain II are boxed in black, red, blue, and violet, respectively. **(B)** Multiple alignment of the deduced amino acid sequence of SmAREB1 and the counterpart protein from other plants. The conserved C1, C2, C3, and C4 domains are boxed in black; the bZIP region is underlined; the NLS domain is boxed in red. The potential five phosphorylation recognition motifs (RXXS/T) are denoted with asterisks (^∗^). All multiple alignments were performed using DNAMAN. **(C)** The positions and length of exons and introns of *SmSnRK2.3*, *SmSnRK2.6*, and *SmAREB1* are displayed schematically. Rounded rectangles indicate exons, while black lines indicate introns.

**FIGURE 2:**
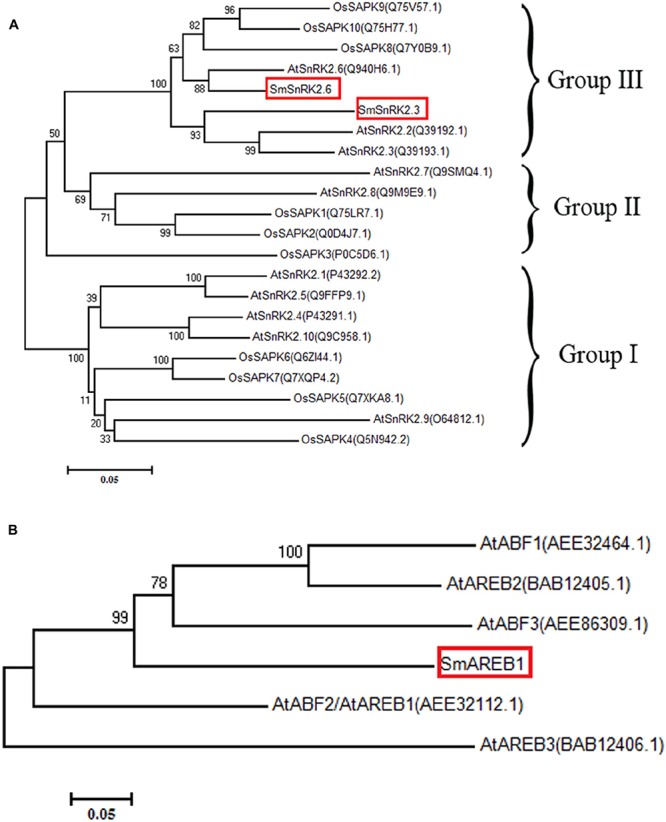
Phylogenetic analysis of *SmSnRK2.3*, *SmSnRK2.6*, and *SmAREB1.* **(A)** A phylogenetic tree constructed based on the amino acid sequences of SmSnRK2.3/2.6 (boxed in red) and SnRK2 family proteins from *A. thaliana* (At) and *O. Sativa* (Os). **(B)** Phylogenetic tree, constructed based on the amino acid sequences of SmAREB1 (boxed in red) and its orthologs in *A. thaliana* (At). These phylogenetic trees were constructed via MEGA7.0, using the neighbor-joining method with 1,000 bootstrap replicates.

*SmSnRK2.3* contained a 1,068 bp ORF, encoding a protein of 355 amino acids with a predicted molecular mass of 40.327 kDa. *SmSnRK2.6* contained a 1,098 bp ORF, encoding a protein of 365 amino acids with a predicted molecular mass of 41.340 kDa. The amino acid sequence analysis and multiple alignments revealed that both *SmSnRK2.3* and *SmSnRK2.6* had a highly conserved N-terminal kinase domain, in addition to C-terminal regulatory domain that consisted of domains I and II (**Figure 1A**). To visually examine their evolutionary origins, the two SmSnRK2s, along with 10 SnRK2 proteins from *A. thaliana* and 10 SAPK proteins from *O. sativa*, were used to construct a phylogenetic tree. As shown in **Figure 2A**, both SmSnRK2.3 and SmSnRK2.6 were clustered within subclass III of the SnRK2. However, SmSnRK2.3 showed a higher degree of similarity with AtSnRK2.3 than with AtSnRK2.2. SmSnRK2.6 shared the same branch and had the highest degree of similarity with AtSnRK2.6. Hence, we named these two genes *SmSnRK2.3* and *SmSnRK2.6*.

*SmAREB1* contained a 1,272 bp ORF, encoding 423 deduced amino acid residues with a calculated molecular mass of 45.467 kDa. This protein has a characteristic basic region-leucine zipper (bZIP) domain and a putative nuclear localization signal (NLS), both located at the C-terminal region. In addition, it had four conserved domains (C1, C2, C3, and C4) very similar to the four conserved domains of AtAREB1 (**Figure 1B**). In AtAREB1, the Ser/Thr residues at RXXS/T in the conserved C1, C2, and C3 regions were phosphorylated by the SnRK2-type protein kinases (Furihata et al., 2006). SmAREB1 shares a 73% identity to the counterpart protein of *Sesamum indicum* (XP_011088250.1), 63% of *Erythranthe guttata* (XP_012836939.1), 59% of *N. tabacum* (XP_016458525.1), and 57% of *Solanum lycopersicum* (XP_004230778.1) (**Figure 1B**). The phylogenetic tree illustrates that SmAREB1 shared the same branch with AtAREB1, AtAREB2, and AtABF3 (**Figure 2B**).

Comparing the genomic and cDNA sequences revealed that *SmSnRK2.3* contained seven introns and eight exons; the *SmSnRK2.6* gene contained eight introns and nine exons; and *SmAREB1* contained three introns and four exons (**Figure 1C**).

### Isolation and Analysis of Promoters

The promoter sequences of *SmSnRK2.3*, *SmSnRK2.6*, and *SmAREB1* were identified via cloning and sequencing. The PlantCARE analysis showed that they contained several *cis*-elements which are related to phytohormone response, abiotic and biotic stresses, and plant development, with the exception of core *cis*-acting elements, such as the TATA box and CAAT box (**Table 1**). Moreover, more than two ABREs were detected in all of their promoters, likely because the expression of ABA responsive genes requiring multiple ABREs or the combination of an ABRE with one of several coupling elements (Fujita et al., 2013). These results indicated that all the three genes may be involved in the *S. miltiorrhiza* responses to ABA and stresses.

**Table 1:** Potential *cis*-acting regulatory elements in the *SmSnRK2.3, SmSnRK2.6,* and *SmAREB1* promoter with the exception of core *cis*-acting elements (TATA box and CAAT box).

*Cis*-element	Number of *cis*-elements			Function
	SmSnRK2.3	SmSnRK2.6	SmAREB1	
ABRE	2	4	3	*cis*-acting element involved in the abscisic acid responsiveness
G-box/G-Box	4	8	7	*cis*-acting regulatory element involved in light responsiveness
MBS	4	2	1	MYB Binding Site involved in drought-inducibility
TC-rich repeats	1		3	*cis*-acting element involved in defense and stress responsiveness
AuxRR-core/TGA-element	2		2	Auxin-responsive element
TGACG-motif/CGTCA-motif	16	4	4	*cis*-acting regulatory element involved in the MeJA-responsiveness
HSE		2	3	*cis*-acting element involved in heat stress responsiveness
P-box/TATC-box/GARE-motif	2	1	2	*cis*-acting element involved in gibberellin-responsiveness
TCA-element	3	5	2	*cis*-acting element involved in salicylic acid responsiveness
WUN-motif			1	Wound-responsive element
ERE		1		Ethylene-responsive element
BoX-W1		1		Fungal elicitor responsive element
LTR	3	3		*cis*-acting element involved in low-temperature responsiveness
Skn-1_motif/GCN4_motif	3	5	1	*cis*-acting regulatory element required for endosperm expression

### Expression Analysis of Genes in *S. miltiorrhiza* Tissues and in Response to ABA

**FIGURE 3:**
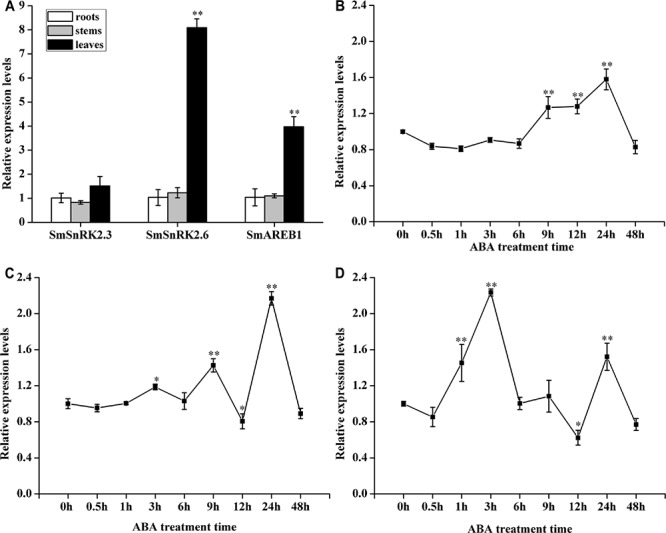
Expression patterns of *SmSnRK2.3*, *SmSnRK2.6*, and *SmAREB1* in various tissues and in response to exogenous ABA treatment. **(A)** Expression patterns of *SmSnRK2.3*, *SmSnRK2.6*, and *SmAREB1* in different tissues of *S. miltiorrhiza.* The expression levels in roots were arbitrarily set to 1 as control. **(B–D)** Expression patterns of *SmSnRK2.3*, *SmSnRK2.6*, and *SmAREB1* under exogenous ABA treatment. The expression levels at 0 h were arbitrarily set to 1 as control. Data represent means ± SD of three replicates. Asterisks denote significant differences compared to control samples at ^∗^*P* < 0.05, ^∗∗^*P* < 0.01, according to Tukey’s test.

As **Figure 3A** shows, these three genes were ubiquitously expressed in the roots, stems, and leaves of *S. miltiorrhiza*. No clear differences were found in the expression levels of *SmSnRK2.3* among the tissues. However, the expression levels of *SmSnRK2.6* and *SmAREB1* in the leaves were significantly higher (*p* < 0.01) than those in the roots and stems, while that in the roots and stems were very similar.

As **Figure 3B** shows, when ABA treatment began, the expression level of *SmSnRK2.3* declined slightly and then remained steady until 6 h, after which it rose significantly (*p* < 0.01), peaking at 24 h, followed by sharp decrease to a minimum at 48 h. A similar pattern was seen under the ABA treatment, in that after a brief and slight decline at 0.5 h, the expression of *SmSnRK2.6* and *SmAREB1* began to increase, peaking at 3 h, but then declined sharply to a minimum at 12 h (with the exception of a small rise at 9 h), followed by another significant peak (*p* < 0.01) at 24 h before it declining again at 48 h (**Figures 3C,D**).

### Subcellular Localization of *SmSnRK2.3*, *SmSnRK2.6*, and *SmAREB1*

**FIGURE 4:**
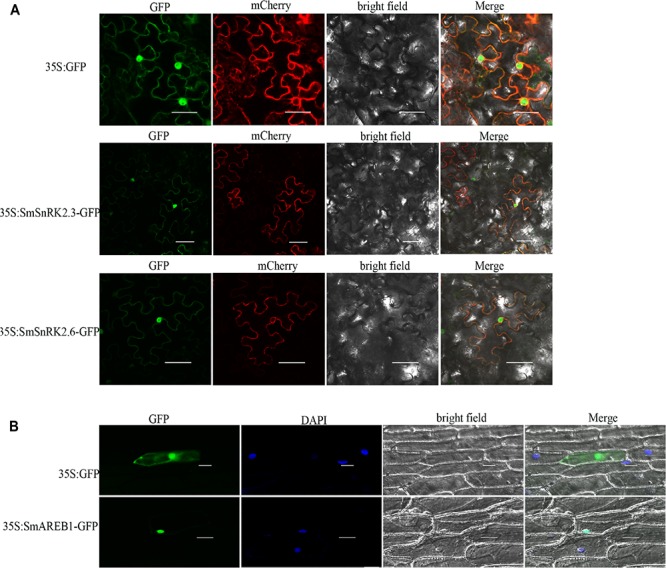
Subcellular localization of *SmSnRK2.3*, *SmSnRK2.6*, and *SmAREB1.* **(A)** Subcellular localization of *SmSnRK2.3* and *SmSnRK2.6* in leaf epidermal cells of tobacco. GFP: green fluorescence; mCherry: red fluorescence of plasma membrane marker; Merge: merge of bright field and relevant fluorescences. Scale bar = 50 μm. **(B)** Subcellular localization of *SmAREB1* in onion epidermal cells. GFP, green fluorescence; DAPI, fluorescence of DAPI nuclear dye; Merge, merge of bright field, GFP, and DAPI. Independent of **(A)** or **(B)**, fluorescences of the empty vector pCAMBIA1301 were used as control. Scale bar = 50 μm.

A detailed investigation of the subcellular distribution of target proteins should enhance our understanding of their functions. As shown in **Figure 4A**, the *N. benthamiana* epidermal cells, which were infected by *A. tumefaciens* and harbored the empty vector pCAMBIA1301, showed a ubiquitous fluorescent distribution. However, both *SmSnRK2.3*-p1301 and *SmSnRK2.6*-p1301 fusion proteins generated GFP signals not only in the cell membrane, since it overlapping with the plasma membrane marker, but also in the cytoplasm and nucleus of the *N. benthamiana* epidermal cells. These results indicated that *SmSnRK2.3* and *SmSnRK2.6* were located in the cell membrane, cytoplasm, and nucleus, which are consistent with the subcellular localization of *TaSnRK2.4/2.7/2.8*, and *ZmSAPK8* (Mao et al., 2010; Zhang H. et al., 2010; Ying et al., 2011; Zhang et al., 2011). The *SmAREB1*-p1301 fusion protein was localized in the nucleus exclusively, since overlapping with the DAPI nuclear dye (**Figure 4B**).

### Transcriptional Activation Analysis of *SmAREB1*

**FIGURE 5:**
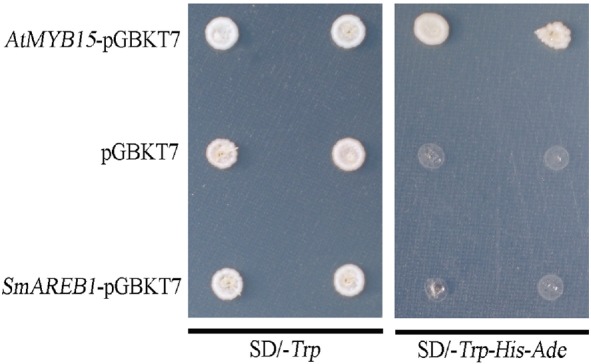
Transactivation activity of *SmAREB1* in yeast. Yeast cells carrying *AtMYB15*-pGBKT7 (positive control), *SmAREB1-*pGBKT7, or pGBKT7 (negative control) were spotted onto SD/-*Trp* and SD/*-Trp-His-Ade* plates, cultured at 30°C for 3 days, respectively.

The AH109 yeast cells containing pGBKT7, *AtMYB15*-pGBKT7, and *SmAREB1*-pGBKT7 grew well on the SD/-Trp medium. However, on SD/-Trp-His-Ade medium, the yeast cells harboring both the *SmAREB1*-pGBKT7 and negative control pGBKT7 constructs were unable to grow. By contrast, those cells harboring the positive control, *AtMYB15*-pGBKT7, did grow well (**Figure 5**). Together, these results demonstrated that SmAREB1 did not activate transcription in the AH109 yeast cells.

### Physical Interaction between SmSnRK2.3/2.6 and SmAREB1

**FIGURE 6:**
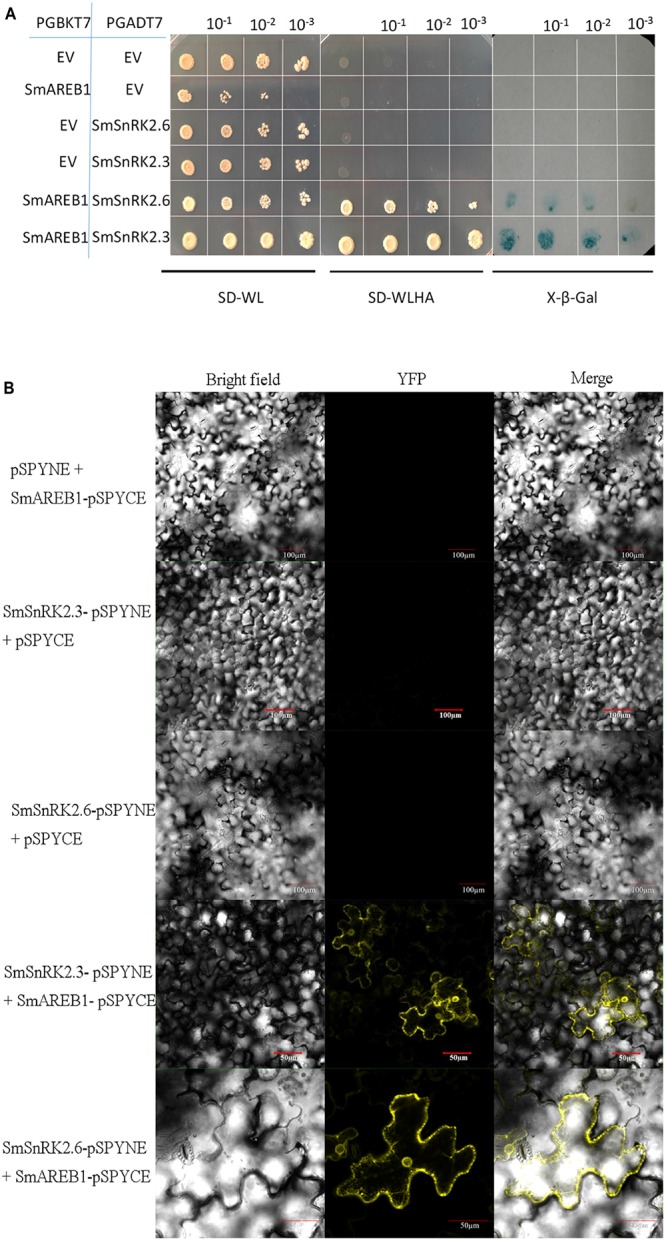
Interaction of *SmSnRK2.3/2.6* with *SmAREB1.* **(A)** Yeast two-hybrid assay of the interaction between *SmSnRK2.3/2.6* and *SmAREB1*. AH109 yeast cells harbor the indicated plasmid combinations, which were orderly diluted 10, 100, and 1000-fold, cultured on SD/-Trp-Leu medium and SD/-Trp-Leu-His-Ade medium, and followed by X-β-Gal staining. **(B)** Bimolecular fluorescence complementation (BiFC) assay of the interactions between *SmSnRK2.3/2.6* and *SmAREB1*. YFP, yellow fluorescence; Merge, merge of bright field and YFP.

To investigate whether the protein kinase SmSnRK2.3/2.6 interacts with the SmAREB1 transcription factor, Y2H and BiFc assays were simultaneously utilized for *in vitro* and *in vivo* verification. Yeast cells co-transformed by *SmSnRK2.3/2.6*-AD + *SmAREB1*-BD not only grew well on SD/-Trp-Leu medium, but also grew on the SD/-Trp-Leu-His-Ade medium, in addition to their ability to turn blue in the X-β-Gal staining assay. However, all the yeast cells that harbored the negative controls could only grow on the SD/-Trp-Leu medium, but not on SD/-Trp-Leu-His-Ade medium (**Figure 6A**). The BiFc analysis revealed strong YFP fluorescent signals in the *N. benthamiana* leaf epidermal cells infected by the *A. tumefaciens* strain EHA105, which harbored the *SmSnRK2.3/2.6*-pSPYNE + *SmAREB1*-pSPYCE plasmids. However, no YFP fluorescent signals were observed in any of the negative controls (**Figure 6B**). In Combination, these analyses indicated that the SmSnRK2.3/2.6 protein could interact with the SmAREB1 transcription factor.

### Protein Purification and LC-MS/MS Analysis of SmSnRK2.3/2.6

Following the Coomassie Brilliant Blue R250 staining, the SDS-PAGE results revealed that the molecular weights of the SmSnRK2.3/2.6 (fused with GST-tag protein) agreed with their previously predicted size (including the 26 kDa of the GST-tag protein) (Supplementary Figures S1A, 2A). Through the induced expression and ultrasonic breaking of the *E. coli* cells, purified proteins were obtained, under native conditions without denaturation, which could be verified via Western blotting (Supplementary Figures S1B, 2B), and analyzed by the LC-MS/MS system.

The LC-MS/MS analysis revealed that this method achieved a 37.78% sequence coverage of the SmSnRK2.3 fusion protein, which was almost half that of SmSnRK2.6 (73.15%). Accordingly, we found several phosphorylated peptides and 14 non-redundant phosphorylation sites in SmSnRK2.6, but only 3 non-redundant phosphorylation sites were found in SmSnRK2.3 (**Table 2**).

**Table 2:** The non-redundant phosphorylated peptides and phosphorylation sites in SmSnRK2.3/2.6 identified by LC-MS/MS.

Fusion protein	Peptide	Phosphorylation site	Score
SmSnRK2.6	K.ICDFGYSKS#S#VLHS#QPKS#TVGTPAYIAPEVLLK.K	Ser-170,Ser-171, Ser-175,Ser-179	49.32
SmSnRK2.6	R.DIGS#GNFGVAR.L	Ser-33	66.81
SmSnRK2.6	V.RFKEVILT#PT#HLAIVMEYASGGELFER.I	Thr-90,Thr-92	48.15
SmSnRK2.6	R.FFFQQLISGVS#YCHAMQICHR.D	Ser-149	100.1
SmSnRK2.6	R.LKICDFGYS#K.S	Ser-168	60.08
SmSnRK2.6	K.SS#VLHSQPKST#VGTPAYIAPEVLLKK.E	Ser-171,Thr-180	43.41
SmSnRK2.6	K.STVGT#PAYIAPEVLLKK.E	Thr-183	47.57
SmSnRK2.6	K.T#IQRILNVQYSIPDYVHISPECR.H	Thr-233	57.68
SmSnRK2.6	R.ILNVQYS#IPDYVHISPECR.H	Ser-243	67.77
SmSnRK2.6	K.RIS#IPEIKNHEWFLK.N	Ser-271	42.63
SmSnRK2.3	R.DIGS#GNFGVAR.L	Ser-31	32.74
SmSnRK2.3	R.S#LRHPNIVR.F	Ser-73	21.31
SmSnRK2.3	K.ST#VGTPAYIAPEVLHR.K	Thr-178	71.03

### Effects of *SmSnRK2.3*, *SmSnRK2.6*, and *SmAREB1* Overexpression on the Content of Phenolic Acids

**FIGURE 7:**
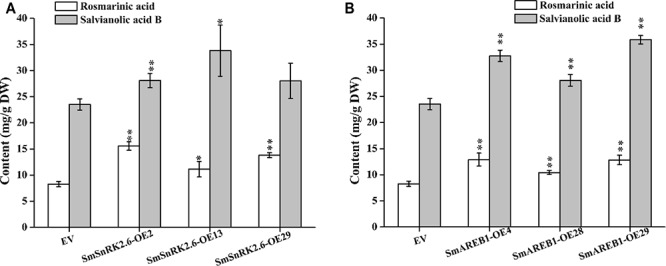
Analysis of RA and Sal B contents in transgenic hairy root lines and empty vector control lines (EV). **(A)** Contents of RA and Sal B in transgenic lines over-expressing *SmSnRK2.6 (SmSnRK2.6-OEs)* and EV. **(B)** Contents of RA and Sal B in transgenic lines over-expressing *SmAREB1 (SmAREB1-OEs)* and EV. Values are mean ± SD, *n* = 3. Asterisks indicate significant differences between transgenic lines and EV control, obtained via Student’s *t*-test (^∗^*P* < 0.05, ^∗∗^*P* < 0.01).

In this study, positive transgenic hairy root lines overexpressing *SmSnRK2.3*, *SmSnRK2.6*, and *SmAREB1* (i.e., *SmSnRK2.3*-OEs, *SmSnRK2.6*-OEs, and *SmAREB1*-OEs) were obtained and identified via PCR, to evaluate the contribution of *SmSnRK2.3/2.6* and *SmAREB1* to the regulation of the phenolic acid metabolism in *S. miltiorrhiza*. The contents of Sal B and RA in the *SmSnRK2.3-*OEs were apparently very similar to those in the empty-vector control lines (EVs) (results not shown). However, we found a remarkable increase (*p* < 0.05) of Sal B and RA contents in *SmSnRK2.6-*OE13, and these contents were significantly enhanced (*p* < 0.01) in *SmSnRK2.6-*OE2, *SmAREB1-*OE4, *SmAREB1-*OE28, and *SmAREB1-*OE29, when compared with those in the EVs. In *SmSnRK2.6-*OE29, whereas only the RA content was significantly higher (*p* < 0.01), the Sal B content did not obviously increase (*p >* 0.05) relative to the EVs (**Figures 7A,B**).

### Effects of *SmSnRK2.6* and *SmAREB1* Overexpression on Expression Levels of Structural Genes

In this study, *SmSnRK2.6*-OE2, *SmSnRK2.6*-OE13, *SmAREB1*-OE28, and *SmAREB1*-OE29 were selected as representatives to investigate the molecular mechanism enabling *SmSnRK2.6* and *SmAREB1* overexpression to increase the contents of phenolic acids in the *S. miltiorrhiza* hairy roots.

The qRT-PCR was performed with two aims: First, to analyze the expression level of structural enzyme genes, namely phenylalanine ammonia lyase 1 (*SmPAL1*), cinnamic acid 4-hydroxylase (*SmC4H*), 4-coumaric acid CoA-ligase 1 (*Sm4CL1*), tyrosine aminotransferase (*SmTAT*), 4-hydroxyphenylpyruvate reductase (*SmHPPR*), and RA synthase (*SmRAS*), which participate in the phenolic acid biosynthetic pathway in *S. miltiorrhiza*. Second, to check the expression level of the key enzyme genes located in the entry point to the other side-branch pathways, namely that of chalcone synthase (*SmCHS*, key enzyme in the flavonoid pathway), cinnamoyl-CoA reductase and caffeic acid *O*-methyltransferase (respectively, *SmCCR* and *SmCOMT*, key enzymes in the lignin pathway), in addition to 4-hydroxyphenylpyruvated dioxygenase (*SmHPPD*) which competes for the same substrate as *SmHPPR*.

**FIGURE 8:**
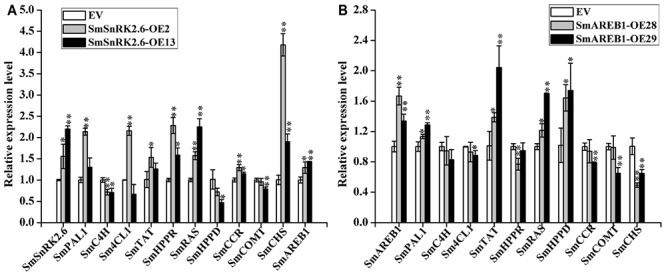
Quantitative real-time PCR analysis of related genes in EV, *SmSnRK2.6-overexpressing* transgenic lines (*SmSnRK2.6-OE2* and *SmSnRK2.6-OE13*) **(A)**, and *SmAREB1-overexpressing* transgenic lines (*SmAREB1-OE28* and *SmAREB1-OE29*) **(B)**. PAL, phenylalanine ammonia lyase; C4H, cinnamic acid 4-hydroxylase; 4CL, 4-coumaric acid CoA-ligase 1; TAT, tyrosine aminotransferase; HPPR, 4-hydroxyphenylpyruvate reductase; RAS, rosmarinic acid synthase; HPPD, 4-hydroxyphenylpyruvate dioxygenase; CHS, chalcone synthase; CCR, cinnamoyl-CoA reductase; COMT, caffeic acid *O*-methyltransferase. Data represent means ± SD of three replicates. Asterisks denote significant differences at ^∗^*P* < 0.05, ^∗∗^*P* < 0.01, compared to the vector control and obtained via Student’s *t*-test.

In both *SmSnRK2.6*-OE2 and *SmSnRK2.6*-OE13, the expression levels of *SmHPPR*, *SmRAS*, *SmCCR*, and *SmCHS* were all significantly increased (*p* < 0.01 or *p* < 0.05), whereas those of *SmC4H* were significantly decreased (*p* < 0.01 or *p* < 0.05). Furthermore, the transcription of *SmPAL1*, *Sm4CL1*, and *SmTAT* in *SmSnRK2.6*-OE2 also significantly increased (*p* < 0.01 or *p* < 0.05), though not in *SmSnRK2.6*-OE13 (*p* > 0.05). In *SmSnRK2.6*-OE2, while the transcription of *SmHPPD* and *SmCOMT* were not significantly changed (*p* > 0.05), in *SmSnRK2.6*-OE13 they were significantly reduced (*p* < 0.05 and *p* < 0.01, respectively) (**Figure 8A**).

In both *SmAREB1*-OE28 and *SmAREB1*-OE29, the expression levels of *SmPAL1*, *SmTAT*, *SmRAS*, and *SmHPPD* were all significantly increased (*p* < 0.05 or *p* < 0.01), while those of *SmC4H*, *Sm4CL1*, *SmCCR*, and *SmCOMT* in *SmAREB1*-OE28 were unchanged (*p >* 0.05), whereas those of *SmHPPR* and *SmCHS* clearly decreased (*p* < 0.01). A different expression pattern was in *SmAREB1*-OE29, in that *SmC4H* and *SmHPPR* levels went unchanged (*p >* 0.05), but those of *Sm4CL1*, *SmCCR*, *SmCOMT*, and *SmCHS* were all significantly reduced (*p* < 0.05 or *p* < 0.01) (**Figure 8B**). Furthermore, the expression levels of *SmAREB1* in *SmSnRK2.6*-OE2 and *SmSnRK2.6*-OE13 were significantly higher (*p* < 0.05 and *p* < 0.01, respectively) than those of the EVs (**Figure 8A**).

## Discussion

ABA plays key regulatory roles in plant growth and development processes, such as seed dormancy and germination, fruit ripening, and stomatal closure (Chen et al., 2016). It is also considered a stress hormone, because its rapid accumulation in plants under stressful conditions can function to protect them against various environmental stresses (Tuteja, 2007). Exogenous ABA treatment could mimic the effects of osmotic stresses and cold stress (Roychoudhury et al., 2013). In our previous work, we found that exogenous ABA could promote the accumulation of phenolic acids in *S. miltiorrhiza* hairy roots (Cui et al., 2012). Increasing evidence shows that the members of subclass III SnRK2 may be strongly activated when treated with ABA, and they have been found to function as the main positive regulators of ABA-dependent signal transduction (Fujii et al., 2011). Therefore, we speculate that members of subclass III SnRK2s in *S. miltiorrhiza* will be good candidate genes for applied use in genetic engineering approaches, whether to improve the ability of *S. miltiorrhiza* to resist stresses or to boost its content of active ingredients, or both.

In this study, we first identified two genes *SmSnRK2.3* and *SmSnRK2.6*, belonging to subclass III of SnRK2, from the *S. miltiorrhiza* hairy roots. PlantCARE analysis showed that their promoters contained ABA and stress response elements (**Table 1**); these results supported the qRT-PCR results, which confirmed that *SmSnRK2.3* and *SmSnRK2.6* were strongly induced by the exogenous ABA treatment (**Figures 3B,C**). Further, the analysis of their amino acid sequences showed that they both contained the myristyl N-terminal myristoylation region (**Figure 1A**), which is essential for protein function in mediating membrane targeting and signal transduction in plant responses to environmental stress (Podell and Gribskov, 2004). Based on the above results, we suggest that both *SmSnRK2.3* and *SmSnRK2.6* are involved in the ABA and stress response of *S. miltiorrhiza*.

Identifying the phosphorylation sites of protein kinases will provide strong evidence for a kinase activity assay, in addition to valuable information for elucidating and understanding the operation of signaling networks based on phosphorylation. The LC-MS/MS analysis of the SmSnRK2.3/2.6-GST fusion protein found 14 non-redundant phosphorylation sites in SmSnRK2.6 and 3 non-redundant sites in SmSnRK2.3 (**Table 2**); a plausible reason for this result may be the low abundance of SmSnRK2.3 protein phosphorylation or a low ionization efficiency of the phosphorylated peptides. Among the identified non-redundant phosphorylation sites in SmSnRK2.3/2.6, we found that Thr178 was in the kinase activation loop of SmSnRK2.3, while Ser168, Ser170, Ser171, Ser175, Ser179, Thr180, and Thr183 were in the kinase activation loop of SmSnRK2.6. The kinase activation loop is reportedly essential for the kinase activity of SnRK2s (Umezawa et al., 2009; Vlad et al., 2010). These results suggest that SmSnRK2.3 and SmSnRK2.6 might be autophosphorylated during recombinant expression and purification, which is an interpretation consistent with other findings (Vlad et al., 2010; Xie et al., 2012).

Substantial evidence also shows that the activated subclass III SnRK2 regulates ABA-responsive gene expression, mainly via phosphorylation of the AREB/ABFs under osmotic stress conditions. AREB/ABFs belong to the group A bZIP transcription factors, which bind to the conserved *cis*-element ABRE within the promoters of many ABA-induced genes to activate their transcription (Choi et al., 2000; Uno et al., 2000). In this study, we also isolated and characterized an AREB subfamily member, *SmAREB1*, which was strongly induced by the exogenous ABA treatment (**Figure 3D**). *SmAREB1* was deemed a characteristic bZIP transcription factor, based on the results of our amino acid sequence analysis (**Figure 1B**) and subcellular localization assay of *SmAREB1* (**Figure 4B**). However, *SmAREB1* had no activity in the transcriptional activation assay (**Figure 5**). According to literature, the activation of AtAREB1 reportedly requires the ABA-dependent posttranscriptional phosphorylation of Ser/Thr residues in the AtAREB1 conserved regions (Furihata et al., 2006). Given that SmAREB1 contains four conserved domains very similar to those of AtAREB1 (**Figure 1B**), we speculated that the SmAREB1 protein requires phosphorylation by SnRK2 family proteins, or it depends upon regulation by other proteins to perform its transcriptional regulation. The result that the SmSnRK2.3/2.6 protein interacted with the SmAREB1 protein (**Figures 6A,B**) lends support to this conjecture: i.e., SmSnRK2.3/2.6 might directly phosphorylate SmAREB1 to “switch on” the activity of SmAREB1. This phosphorylation role still requires validation and could be verified through an in-gel kinase assay in future research.

Furthermore, we investigated whether or not *SmSnRK2.3*, *SmSnRK2.6*, and *SmAREB1* participated in the regulation of phenolic acid synthesis. To do this, overexpression vectors of *SmSnRK2.3/2.6* and *SmAREB1* were built under the control of the CaMV35S promoter to obtain *SmSnRK2.3*-OEs, *SmSnRK2.6*-OEs, and *SmAREB1*-OEs. In this respect, the hairy root system we used is considered optimal for studying the metabolic regulation of effective components in *S. miltiorrhiza*, as it has the following advantages: high-level productivity of secondary metabolites, stable hereditary, rapid growth under simple conditions, and inclusion of the characteristic secondary metabolic pathway of the parent plants (Chen et al., 1999; Guillon et al., 2006; Kai et al., 2011).

Overexpression of *SmSnRK2.3* did not significantly increase the contents of Sal B and RA, whereas overexpression of *SmSnRK2.6* and *SmAREB1* significantly increased both (**Figures 7A,B**). Two plausible explanations for these results are as follows: (1) The qRT-PCR analyses revealed that *SmSnRK2.3* was ubiquitously expressed in roots, stems, and leaves of *S. miltiorrhiza*, but to the same degree in all tissues. However, the greatest expression levels of *SmSnRK2.6* and *SmAREB1* were detected in the leaves. A larger accumulation of phenolic acids has been reported in *S. miltiorrhiza* leaves (Hang et al., 2008). So overexpression of *SmSnRK2.6* and *SmAREB1* are more conducive to promote substantial accumulation of phenolic acids, compared to that of *SmSnRK2.3.* (2) The LC-MS/MS analysis of the SmSnRK2.3/2.6-GST fusion protein indicated that SmSnRK2.3 had a lower phosphorylated abundance, when compared with SmSnRK2.6, which suggests that SmSnRK2.3 cannot effectively activate SmAREB1 to regulate downstream gene expression. As a drought-sensitive plant species, *S. miltiorrhiza* resists oxidative stress via the accumulation of phenolic acids (Bettaieb et al., 2011). Therefore, we speculate that the overexpression of *SmSnRK2.6* and *SmAREB1* would increase the contents of phenolic acids, thus enhancing the antioxidant activity of *S. miltiorrhiza* (leading to an increased tolerance of osmotic stresses).

It has been reported that rosmarinic acid synthase (RAS) is the most specific enzyme in the phenolic acid biosynthetic pathway, because it couples products from the phenylpropanoid and tyrosine-derived pathways (Di et al., 2013; Wang et al., 2015). Irrespective of how the expression levels were regulated of the other structural genes participating in phenolic acid-branched and side-branched pathways, the RAS transcripts were significantly enhanced in the *SmSnRK2.6*-OEs and *SmAREB1*-OEs. Our qRT-PCR results revealed that the overexpression of *SmSnRK2.6* and *SmAREB1* ultimately promoted more metabolic flux to the phenolic acid-branched pathway, by regulating the expression levels of structural enzyme genes that participate in the phenolic acid-branched and competition bypass-branched pathways (**Figures 8A,B**). Besides, the expression levels of *SmCHS* in the *SmSnRK2.6*-OEs were significantly higher than those in the EVs. *SmCHS* is the key enzyme in the entry point to the flavonoid pathway; these results indicate that an overexpression of *SmSnRK2.6* might also promote the accumulation of flavonoids, which have long been recognized as playing multiple roles in the responses of higher plants to a wide range of environmental stresses (Agati and Tattini, 2010). Prior studies reported the existence of ABRE *cis*-acting elements in the promoters of key enzyme genes involved in the biosynthetic pathway of phenolic acid (Huang et al., 2008; Song et al., 2012). In the future, we plan to conduct electrophoretic mobility shift assay (EMSA) and other experiments to verify whether *SmAREB1* regulates the expression of these key enzyme genes by directly binding to the ABRE *cis*-acting elements in their promoter regions.

## Conclusion

Three novel genes named *SmSnRK2.3*, *SmSnRK2.6*, and *SmAREB1* were cloned from *S. miltiorrhiza* hairy roots and functionally characterized. Comparing to *SmSnRK2.3*, overexpression of *SmSnRK2.6* significantly promotes the accumulation of RA and Sal B in the *S. miltiorrhiza* hairy roots, by effectively regulating the transcription activity of *SmAREB1*. Moreover, it is suggested that *SmSnRK2.6* was involved in ABA and stress response of *S. miltiorrhiza*. Therefore, this study provides one candidate gene, SmSnRK2.6, for breeding transgenic *S. miltiorrhiza* lines featuring improved tolerance to abiotic stresses and increased active ingredients. Furthermore, these results provide a theoretical foundation to elucidate the molecular mechanism underlying the ability of exogenous ABA to increase the content of phenolic acids in *S. miltiorrhiza* hairy roots.

## Accession Numbers

The cDNA sequences of *SmSnRK2.3*, *SmSnRK2.6*, and *SmAREB1* in *S. miltiorrhiza* cloned in this study were deposited in GenBank under the accession No. MF185314, No. MF185316, and No. MF185317.

## Author Contributions

ZL designed the research and led this project. YJ, ZB, TP, and KD carried out the experiments and analyzed the results. YJ wrote the manuscript. YG provided scientific advice and revised the manuscript. All authors have read and approved the final manuscript.

## Conflict of Interest Statement

The authors declare that the research was conducted in the absence of any commercial or financial relationships that could be construed as a potential conflict of interest.

## Footnotes

1 https://www.ncbi.nlm.nih.gov/orffinder/

2 http://genes.mit.edu/GENSCAN.html

3 http://web.expasy.org/protparam/

4 https://blast.ncbi.nlm.nih.gov/Blast.cgi

5 http://hits.isb-sib.ch/cgi-bin/PFSCAN

6 http://www.ncbi.nlm.nih.gov

7 http://gsds.cbi.pku.edu.cn/

8 http://bioinformatics.psb.ugent.be/webtools/plantcare/html/

## References

[B1] Agati G. Tattini M. (2010). Multiple functional roles of flavonoids in photoprotection. *New Phytol.* 186 786–793. 20569414 10.1111/j.1469-8137.2010.03269.x

[B2] Akula R. (2011). Influence of abiotic stress signals on secondary metabolites in plants. *Plant Signal. Behav.* 6 1720–1731. 10.4161/psb.6.11.17613 22041989 PMC3329344

[B3] Bettaieb I. Hamrouni-Sellami I. Bourgou S. Limam F. Marzouk B. (2011). Drought effects on polyphenol composition and antioxidant activities in aerial parts of *Salvia officinalis* L. *Acta Physiol. Plant.* 33 1103–1111. 10.1007/s11738-010-0638-z

[B4] Chen H. Chen F. Zhang Y. L. Song J. Y. (1999). Production of lithospermic acid B and rosmarinic acid in hairy root cultures of *Salvia miltiorrhiza*. *J. Ind. Microbiol. Biotechnol.* 22 133–138. 10.1038/sj.jim.2900624

[B5] Chen H. Chena F. Chiu F. C. K. Lo C. M. Y. (2001). The effect of yeast elicitor on the growth and secondary metabolism of hairy root cultures of *Salvia miltiorrhiza*. *Enzyme Microb. Technol.* 28 100–105. 10.1016/S0141-0229(00)00284-2 11118603

[B6] Chen P. Sun Y. F. Kai W. B. Liang B. Zhang Y. S. Zhai X. W. (2016). Interactions of ABA signaling core components (SlPYLs, SlPP2Cs, and SlSnRK2s) in tomato (*Solanum lycopersicon*). *J. Plant Physiol.* 205 67–74. 10.1016/j.jplph.2016.07.016 27626883

[B7] Choi H. Hong J. Ha J. Kang J. Kim S. Y. (2000). ABFs, a family of ABA-responsive element binding factors. *J. Biol. Chem.* 275 1723–1730. 10.1074/jbc.275.3.1723 10636868

[B8] Cui B. Liang Z. Liu Y. Liu F. Zhu J. (2012). Effects of ABA and its biosynthetic inhibitor fluridone on accumulation of penolic acids and activity of PAL and TAT in hairy root of *Salvia miltiorrhiza*. *China J. Chin. Mater. Med.* 37 754–759. 22715716

[B9] Di P. Zhang L. Chen J. Tan H. Xiao Y. Dong X. (2013). ^13^C tracer reveals phenolic acids biosynthesis in hairy root cultures of *Salvia miltiorrhiza*. *ACS Chem. Biol.* 8 1537–1548. 10.1021/cb3006962 23614461

[B10] Fujii H. Verslues P. E. Zhu J. K. (2011). *Arabidopsis* decuple mutant reveals the importance of SnRK2 kinases in osmotic stress responses in vivo. *Proc. Natl. Acad. Sci. U.S.A.* 108 1717–1722. 10.1073/pnas.1018367108 21220313 PMC3029766

[B11] Fujii H. Zhu J. K. (2009). Arabidopsis mutant deficient in 3 abscisic acid-activated protein kinases reveals critical roles in growth, reproduction, and stress. *Proc. Natl. Acad. Sci. U.S.A.* 106 8380–8385. 10.1073/pnas.0903144106 19420218 PMC2688869

[B12] Fujita Y. Yoshida T. Yamaguchi-Shinozaki K. (2013). Pivotal role of the AREB/ABF-SnRK2 pathway in ABRE-mediated transcription in response to osmotic stress in plants. *Physiol. Plant.* 147 15–27. 10.1111/j.1399-3054.2012.01635.x 22519646

[B13] Furihata T. Maruyama K. Fujita Y. Umezawa T. Yoshida R. Shinozaki K. (2006). Abscisic acid-dependent multisite phosphorylation regulates the activity of a transcription activator AREB1. *Proc. Natl. Acad. Sci. U.S.A.* 103 1988–1993. 10.1073/pnas.0505667103 16446457 PMC1413621

[B14] Guillon S. Tremouillaux-Guiller J. Pati P. K. Rideau M. Gantet P. (2006). Hairy root research: recent scenario and exciting prospects – Commentary. *Curr. Opin. Plant Biol.* 9 341–346. 10.1016/j.pbi.2006.03.008 16616871

[B15] Hang L. Wang J. Yang D. Shu Z. Liang Z. (2008). Distribution traits of bioactives in different parts of *Salvia miltiorrhiza* Bunge. and *Salvia miltiorrhiza* Bunge. f. alba. *J. Northwest Agric. For. Univ.* 36 217–222.

[B16] Hao G. Jiang X. Feng L. Tao R. Li Y. Huang L. (2016). Cloning, molecular characterization and functional analysis of a putative R2R3-MYB transcription factor of the phenolic acid biosynthetic pathway in *S. miltiorrhiza* Bge. f. alba. *Plant Cell Tissue Organ Cult.* 124 151–168. 10.1007/s11240-015-0883-3

[B17] Hu B. Jin J. Guo A.-Y. Zhang H. Luo J. Gao G. (2015). GSDS 2.0: an upgraded gene feature visualization server. *Bioinformatics* 31 1296–1297. 10.1093/bioinformatics/btu817 25504850 PMC4393523

[B18] Hu W. E. I. Huang C. Deng X. Zhou S. Chen L. Li Y. (2013). TaASR1 a transcription factor gene in wheat, confers drought stress tolerance in transgenic tobacco. *Plant Cell Environ.* 36 1449–1464. 10.1111/pce.12074 23356734

[B19] Huang B. Yi B. Duan Y. Sun L. Yu X. Guo J. (2008). Characterization and expression profiling of tyrosine aminotransferase gene from *Salvia miltiorrhiza* (Dan-shen) in rosmarinic acid biosynthesis pathway. *Mol. Biol. Rep.* 35 601–612. 10.1007/s11033-007-9130-2 17805988

[B20] Kai G. Xu H. Zhou C. Liao P. Xiao J. Luo X. (2011). Metabolic engineering tanshinone biosynthetic pathway in *Salvia miltiorrhiza* hairy root cultures. *Metab. Eng.* 13 319–327. 10.1016/j.ymben.2011.02.003 21335099

[B21] Lescot M. Dehais P. Thijs G. Marchal K. Moreau Y. Van de Peer Y. (2002). PlantCARE, a database of plant cis-acting regulatory elements and a portal to tools for in silico analysis of promoter sequences. *Nucleic Acids Res.* 30 325–327. 10.1093/nar/30.1.325 11752327 PMC99092

[B22] Liu H. Wang X. Wang D. Zou Z. Liang Z. (2011). Effect of drought stress on growth and accumulation of active constituents in *Salvia miltiorrhiza* Bunge. *Ind. Crop Prod.* 33 84–88. 10.1016/j.indcrop.2010.09.006

[B23] Liu L. Yang D. Liang T. Zhang H. He Z. Liang Z. (2016). Phosphate starvation promoted the accumulation of phenolic acids by inducing the key enzyme genes in *Salvia miltiorrhiza* hairy roots. *Plant Cell Rep.* 35 1933–1942. 10.1007/s00299-016-2007-x 27271760

[B24] Liu Y. Sun G. Zhong Z. Ji L. Zhang Y. Zhou J. (2016). Overexpression of AtEDT1 promotes root elongation and affects medicinal secondary metabolite biosynthesis in roots of transgenic *Salvia miltiorrhiza*. *Protoplasma* 254 1617–1625. 10.1007/s00709-016-1045-0 27915455

[B25] Livak K. J. Schmittgen T. D. (2001). Analysis of relative gene expression data using real-time quantitative PCR and the 2^-ΔΔC_T_^ method. *Methods* 25 402–408. 10.1006/meth.2001.1262 11846609

[B26] Ma P. Liu J. Zhang C. Liang Z. (2013). Regulation of water-soluble phenolic acid biosynthesis in *Salvia miltiorrhiza* Bunge. *Appl. Biochem. Biotechnol.* 170 1253–1262. 10.1007/s12010-013-0265-4 23673485

[B27] Mao X. Zhang H. Tian S. Chang X. Jing R. (2010). TaSnRK2.4 an SNF1-type serine/threonine protein kinase of wheat (*Triticum aestivum* L.), confers enhanced multistress tolerance in *Arabidopsis*. *J. Exp. Bot.* 61 683–696. 10.1093/jxb/erp331 20022921 PMC2814103

[B28] Nelson B. K. Cai X. Nebenfuehr A. (2007). A multicolored set of *in vivo* organelle markers for co-localization studies in Arabidopsis and other plants. *Plant J.* 51 1126–1136. 10.1111/j.1365-313X.2007.03212.x 17666025

[B29] Podell S. Gribskov M. (2004). Predicting N-terminal myristoylation sites in plant proteins. *BMC Genomics* 5:37 10.1186/1471-2164-5-37 PMC449705 15202951

[B30] Roychoudhury A. Paul S. Basu S. (2013). Cross-talk between abscisic acid-dependent and abscisic acid-independent pathways during abiotic stress. *Plant Cell Rep.* 32 985–1006. 10.1007/s00299-013-1414-5 23508256

[B31] Shao Y. Wei J. Wu F. Zhang H. Yang D. Liang Z. (2016). DsTRD: danshen transcriptional resource database. *PLoS ONE* 11:e0149747 10.1371/journal.pone.0149747 PMC4765898 26909679

[B32] Shen X. Zhao K. Liu L. Zhang K. Yuan H. Liao X. (2014). A role for PacMYBA in ABA-regulated anthocyanin biosynthesis in red-colored sweet cherry cv. Hong Deng (*Prunus avium* L.). *Plant Cell Physiol.* 55 862–880. 10.1093/pcp/pcu013 24443499

[B33] Shukla R. K. Raha S. Tripathi V. Chattopadhyay D. (2006). Expression of CAP2 an APETALA2-family transcription factor from chickpea, enhances growth and tolerance to dehydration and salt stress in transgenic tobacco. *Plant Physiol.* 142 113–123. 16844836 10.1104/pp.106.081752 PMC1557594

[B34] Song J. Ji Y. Xu K. Wang Z. (2012). An integrated analysis of the rosmarinic acid-biosynthetic genes to uncover the regulation of rosmarinic acid pathway in *Salvia miltiorrhiza*. *Acta Physiol. Plant.* 34 1501–1511. 10.1007/s11738-012-0948-4

[B35] Tuteja N. (2007). Abscisic acid and abiotic stress signaling. *Plant Signal. Behav.* 2 135–138. 10.4161/psb.2.3.4156 19516981 PMC2634038

[B36] Umezawa T. Sugiyama N. Mizoguchi M. Hayashi S. Myouga F. Yamaguchi-Shinozaki K. (2009). Type 2C protein phosphatases directly regulate abscisic acid-activated protein kinases in *Arabidopsis*. *Proc. Natl. Acad. Sci. U.S.A.* 106 17588–17593. 10.1073/pnas.0907095106 19805022 PMC2754379

[B37] Uno Y. Furihata T. Abe H. Yoshida R. Shinozaki K. Yamaguchi-Shinozaki K. (2000). *Arabidopsis* basic leucine zipper transcription factors involved in an abscisic acid-dependent signal transduction pathway under drought and high-salinity conditions. *Proc. Natl. Acad. Sci. U.S.A.* 97 11632–11637. 10.1073/pnas.190309197 11005831 PMC17252

[B38] Vlad F. Droillard M.-J. Valot B. Khafif M. Rodrigues A. Brault M. (2010). Phospho-site mapping, genetic and in planta activation studies reveal key aspects of the different phosphorylation mechanisms involved in activation of SnRK2s. *Plant J.* 63 778–790. 10.1111/j.1365-313X.2010.04281.x 20561261

[B39] Walter M. Chaban C. Schutze K. Batistic O. Weckermann K. Nake C. (2004). Visualization of protein interactions in living plant cells using bimolecular fluorescence complementation. *Plant J.* 40 428–438. 10.1111/j.1365-313X.2004.02219.x 15469500

[B40] Wang B. Sun W. Li Q. Li Y. Luo H. Song J. (2015). Genome-wide identification of phenolic acid biosynthetic genes in *Salvia miltiorrhiza*. *Planta* 241 711–725. 10.1007/s00425-014-2212-1 25471478

[B41] Wang H. Wu Y. Yang X. Guo X. Cao X. (2017). SmLEA2, a gene for late embryogenesis abundant protein isolated from *Salvia miltiorrhiza*, confers tolerance to drought and salt stress in *Escherichia coli* and *S. miltiorrhiza*. *Protoplasma* 254 685–696. 10.1007/s00709-016-0981-z 27193100

[B42] Wang Z. Cui L. Chen C. Liu X. Yan Y. Wang Z. (2012). Downregulation of cinnamoyl CoA reductase affects lignin and phenolic acids biosynthesis in *Salvia miltiorrhiza* Bunge. *Plant Mol. Biol. Rep.* 30 1229–1236. 10.1007/s11105-012-0444-4

[B43] Wei T. Deng K. Gao Y. Liu Y. Yang M. Zhang L. (2016a). Arabidopsis DREB1B in transgenic *Salvia miltiorrhiza* increased tolerance to drought stress without stunting growth. *Plant Physiol. Biochem.* 104 17–28. 10.1016/j.plaphy.2016.03.003 27002402

[B44] Wei T. Deng K. Liu D. Gao Y. Liu Y. Yang M. (2016b). Ectopic expression of DREB transcription factor, AtDREB1A, confers tolerance to drought in transgenic *Salvia miltiorrhiza*. *Plant Cell Physiol.* 57 1593–1609. 10.1093/pcp/pcw084 27485523

[B45] Wei T. Deng K. Zhang Q. Gao Y. Liu Y. Yang M. (2017). Modulating AtDREB1C expression improves drought tolerance in *Salvia miltiorrhiza*. *Front. Plant Sci.* 8:52 10.3389/fpls.2017.00052 PMC5259653 28174590

[B46] Wisniewski J. R. Zougman A. Nagaraj N. Mann M. (2009). Universal sample preparation method for proteome analysis. *Nat. Methods* 6 359–U360. 10.1038/nmeth.1322 19377485

[B47] Xiao Y. Zhang L. Gao S. Saechao S. Di P. Chen J. (2011). The c4h, tat, hppr and hppd genes prompted engineering of rosmarinic acid biosynthetic pathway in *Salvia miltiorrhiza* hairy root cultures. *PLoS ONE* 6:e29713 10.1371/journal.pone.0029713 PMC3248448 22242141

[B48] Xie T. Ren R. Zhang Y. Y. Pang Y. Yan C. Gong X. (2012). Molecular mechanism for inhibition of a critical component in the *Arabidopsis thaliana* abscisic acid signal transduction pathways, SnRK2.6, by protein phosphatase ABI1. *J. Biol. Chem.* 287 794–802. 10.1074/jbc.M111.313106 22090030 PMC3249133

[B49] Xing B. Yang D. Guo W. Liang Z. Yan X. Zhu Y. (2015). Ag^+^ as a more effective elicitor for production of tanshinones than phenolic acids in *Salvia miltiorrhiza* hairy roots. *Molecules* 20 309–324. 10.3390/molecules20010309 PMC6272699 25547728

[B50] Xu H. Song J. Luo H. Zhang Y. Li Q. Zhu Y. (2016). Analysis of the genome sequence of the medicinal plant *Salvia miltiorrhiza*. *Mol. Plant* 9 949–952. 10.1016/j.molp.2016.03.010 27018390 PMC5517341

[B51] Yan Y.-P. Wang Z. Z. (2007). Genetic transformation of the medicinal plant *Salvia miltiorrhiza* by *Agrobacterium tumefaciens*-mediated method. *Plant Cell Tissue Organ Cult.* 88 175–184. 10.1007/s11240-006-9187-y

[B52] Yang D. Sheng D. Duan Q. Liang X. Liang Z. Liu Y. (2012). PEG and ABA trigger the burst of reactive oxygen species to increase tanshinone production in *Salvia miltiorrhiza* hairy roots. *J. Plant Growth Regul.* 31 579–587. 10.1007/s00344-012-9268-6

[B53] Yang Y. Hou S. Cui G. Chen S. Wei J. Huang L. (2010). Characterization of reference genes for quantitative real-time PCR analysis in various tissues of *Salvia miltiorrhiza*. *Mol. Biol. Rep.* 37 507–513. 10.1007/s11033-009-9703-3 19680786

[B54] Ying S. Zhang D. F. Li H. Y. Liu Y. H. Shi Y. S. Song Y. C. (2011). Cloning and characterization of a maize SnRK2 protein kinase gene confers enhanced salt tolerance in transgenic *Arabidopsis*. *Plant Cell Rep.* 30 1683–1699. 10.1007/s00299-011-1077-z 21638061

[B55] Yoshida T. Fujita Y. Maruyama K. Mogami J. Todaka D. Shinozaki K. (2015). Four *Arabidopsis* AREB/ABF transcription factors function predominantly in gene expression downstream of SnRK2 kinases in abscisic acid signalling in response to osmotic stress. *Plant Cell Environ.* 38 35–49. 10.1111/pce.12351 24738645 PMC4302978

[B56] Yoshida T. Fujita Y. Sayama H. Kidokoro S. Maruyama K. Mizoi J. (2010). AREB1, AREB2, and ABF3 are master transcription factors that cooperatively regulate ABRE-dependent ABA signaling involved in drought stress tolerance and require ABA for full activation. *Plant J.* 61 672–685. 10.1111/j.1365-313X.2009.04092.x 19947981

[B57] Zhang H. Mao X. Jing R. Chang X. Xie H. (2011). Characterization of a common wheat (*Triticum aestivum* L.) TaSnRK2.7 gene involved in abiotic stress responses. *J. Exp. Bot.* 62 975–988. 10.1093/jxb/erq328 21030389 PMC3022395

[B58] Zhang H. Mao X. Wang C. Jing R. (2010). Overexpression of a common wheat gene *TaSnRK2.8* enhances tolerance to drought, salt and low temperature in *Arabidopsis*. *PLoS ONE* 5:e16041 10.1371/journal.pone.0016041 PMC3012728 21209856

[B59] Zhang H. Yang B. Liu W.-Z. Li H. Wang L. Wang B. (2014). Identification and characterization of CBL and CIPK gene families in canola (*Brassica napus* L.). *BMC Plant Biol.* 14:8 10.1186/1471-2229-14-8 PMC3890537 24397480

[B60] Zhang S. Li H. Liang X. Yan Y. Xia P. Jia Y. (2015). Enhanced production of phenolic acids in *Salvia miltiorrhiza* hairy root cultures by combing the RNAi-mediated silencing of chalcone synthase gene with salicylic acid treatment. *Biochem. Eng. J.* 103 185–192. 10.1016/j.bej.2015.07.019

[B61] Zhang S. Ma P. Yang D. Li W. Liang Z. Liu Y. (2013). Cloning and characterization of a putative R2R3 MYB transcriptional repressor of the rosmarinic acid biosynthetic pathway from *Salvia miltiorrhiza*. *PLoS ONE* 8:e73259 10.1371/journal.pone.0073259 PMC3769309 24039895

[B62] Zhang Y. Yan Y. P. Wang Z. Z. (2010). The *Arabidopsis PAP1* transcription factor plays an important role in the enrichment of phenolic acids in *Salvia miltiorrhiza*. *J. Agric. Food Chem.* 58 12168–12175. 10.1021/jf103203e 21058651

[B63] Zhang Y. Yan Y. P. Wu Y. C. Hua W. P. Chen C. Ge Q. (2014). Pathway engineering for phenolic acid accumulations in *Salvia miltiorrhiza* by combinational genetic manipulation. *Metab. Eng.* 21 71–80. 10.1016/j.ymben.2013.10.009 24269612

[B64] Zhao G. Shi Q. Han Y. Li S. Wang C. (2014). The physiological and biochemical responses of a medicinal plant (*Salvia miltiorrhiza* L.) to stress caused by various concentrations of NaCl. *PLoS ONE* 9:e89624 10.1371/journal.pone.0089624 PMC3934908 24586918

