# Supplementary material for: The Protein Kinase SmSnRK2.6 Positively Regulates Phenolic Acid Biosynthesis in Salvia miltiorrhiza by Interacting with SmAREB1
Source: Front Plant Sci. 2017 Aug 9;8:1384. doi: 10.3389/fpls.2017.01384 (PMC5552723; doi:10.3389/fpls.2017.01384)
Supplement: Supplementary file 1 [file Presentation_1.PDF]

Supplementary Material

The Protein Kinase *SmSnRK2.6* Positively Regulates Phenolic acid Biosynthesis in *Salvia miltiorrhiza* by Interacting with *SmAREB1*

Yanyan Jia[a], Zhenqing Bai[a], Tianlin Pei[a], Kai Ding[a], Zongsuo Liang[a,b*] and Yuehua Gong[c*]

[a.] *College of Life Sciences, Northwest A & F University, Yangling 712100, P R China.*

[b.] *College of Life Sciences, Zhejiang Sci-Tech University, Hangzhou 310000, P R China.*

[c.] *Sichuan Tea College, YiBin University, Yibin 644000, P R China.*

* Correspondence: Zongsuo Liang, e-mail: liangzs@ms.iswc.ac.cn; Yuehua Gong, e-mail: gongyh01@163.com

Table S1. Primer sequences used to clone *SmSnRK2.3*, *SmSnRK2.6* and *SmAREB1*

Primer	Sequence (5'-3')
SmSnRK2.3cDNA-F	ATGGATCGGGCAGGAATGACAG
SmSnRK2.3cDNA-R	TCACATTGCATAAATGACCTCC
SmSnRK2.6cDNA-F	TCTCTCTCTGCCGCTCTGGGGAAG
SmSnRK2.6cDNA-R	GCAGTAAAGACAGTGGTAAGATTG
SmAREB1cDNA-F	ATGGGGTCCTACATGAACTTCAAGAAC
SmAREB1cDNA-R	GAGTTACTGCTCTACTCTACCAAGGTC

Table S2. Primers used to amplify promoter of *SmSnRK2.3*, *SmSnRK2.6* and *SmAREB1*

Primer	Sequence (5’-3’)
SmSnRK2.3pro’-F	CACTAATCCTCAGCCATTATC
SmSnRK2.3pro’-R	TTCAGTCTCAAAACCGCCGTC
SmSnRK2.6pro’-F	GAAACTAGAATTATCCATTCACAATAG
SmSnRK2.6pro’-R	CCCGAAATTATCTTC
SmAREB1pro’-F	ACTTGTTATCGTGGACGGAGG
SmAREB1pro’-R	CGTTCCAAGTATGCAAGCAGTGG

Table S3. Primers used in real-time quantitative RT-PCR assays

Primer	Sequence (5'-3')	Primer	Sequence (5'-3')
SmSnRK2.3-F	CCATACCTCCGGCTGGTTTG	SmSnRK2.3-R	CATAAATGACCTCCCCACTGCTG
SmSnRK2.6-F	CAACCCATGCAGAGTGATAGTG	SmSnRK2.6-R	TTTCAAGATCCTCGTCCATGTC
SmAREB1-F	TGGAGAAAGTGGTGGAAAGG	SmAREB1-R	GATTTGCTACTTCTGCTTCGAG
SmACT-F	GGTGCCCTGAGGTCCTGTT	SmACT-R	AGGAACCACCGATCCAGACA
SmUBQ-F	ACCCTCACGGGGAAGACCATC	SmUBQ-R	ACCACGGAGACGGAGGACAAG
SmPAL1-F	GTGAAGAACACCGTGAGCCAG	SmPAL1-R	GTCGTCGATGTAGGCGAAAAC
SmC4H-F	TCTTGCGTTGCCTATTCT	SmC4H-R	CAATGGTCGAGTGCTTCAA
SmTAT-F	AGTAGACGTGCCTGCTCT	SmTAT-R	TGGCTATCCAACTCCTTC
Sm4CL1-F	ATTCGCATTCGCATTTCTCGG	Sm4CL1-R	GCGGCGTAGTGCTTCACCTTT
SmHPPR-F	CCTGACTCCAGAAACAACCCAC	SmHPPR-R	ACCCAGACGACCCTCCACA
SmRAS-F	CAGTTTCCGGTGCCCTAAT	SmRAS-R	TGATGGCGACGAACAAGC
SmHPPD-F	TAGGCCGACCATCTTCATAG	SmHPPD-R	TTCCCGAATCCTCCACAT
SmCCR-F	CTGATGTTGCTTCGCCTTCT	SmCCR-R	CATACGTGCCTTCCCCTTG
SmCOMT-F	GCCACTAAGAATGTTGTCC	SmCOMT-R	TCTGTCCTTTCCTTACCA
SmCHS-F	CGCGATTATGCTTGAGGTTGA	SmCHS-R	CACTACTTGATGTCCCATTTCTTGAC

Sm, Salvia miltiorrhiza. ACT, β-actin; UBQ, Ubiquitin; PAL1, phenylalanine ammonia-lyase 1; C4H, cinnamic acid 4-hydroxylase; TAT, tyrosine aminotransferase; 4CL1, 4-coumaric acid CoA-ligase 1 ; HPPR, 4-hydroxyphenylpyruvate reductase; RAS, rosmarinic acid synthase; HPPD, 4-hydroxyphenylpyruvate dioxygenase ; CCR, cinnamoyl-CoA reductase; COMT, caffeic acid O-methyltransferase; CHS, chalcone synthase

Table S4. Primers used in subcellular localization analysis

Primer	Sequence (5’-3’)
SmSnRK2.3-GFP-F	CGGGATCCATGGATCGGGCAG (*BamH*I site underlined)
SmSnRK2.3-GFP-R	CGGGATCCCATTGCATAAATG (*BamH*I site underlined)
SmSnRK2.6-GFP-F	CGGGATCCATGGATCGACC (*BamH*I site underlined)
SmSnRK2.6-GFP-R	CGGGATCCCATAGCATAGACTATTTC (*BamH*I site underlined)
SmAREB1-GFP-F	CGGGATCCATGGGGTCCTACATGAAC (*BamH*I site underlined)
SmAREB1-GFP-R	CGGGATCCCCAAGGTCCGGTAAGTGT (*BamH*I site underlined)

Table S5. Primers used in Y2H analysis

Primer	Sequence (5'-3')
SmSnRK2.3-AD-F	CGGAATTCATGGATCGGGCAG (*EcoR*I site underlined)
SmSnRK2.3-AD-R	CGGGATCCCTCACATTGCATAAATGAC (*BamH*I site underlined)
SmSnRK2.6-AD-F	CGGAATTCATGGATCGACCGCCGGTCAC (*EcoR*I site underlined)
SmSnRK2.6-AD-R	CGGGATCCCTCACATAGCATAGAC (*BamH*I site underlined)
SmAREB1-BD-F	CGGAATTCATGGGGTCCTAC (*EcoR*I site underlined)
SmAREB1-BD-R	CGGGATCCCCTACCAAGGTC (*BamH*I site underlined)

Table S6. Primers used in construction of the SmSnRK2.3/2.6-GST fusion vectors

Primer	Sequence (5’-3’)
SmSnRK2.3-GST-F	CGGGATCCATGGATCGGGCAG (*BamH*I site underlined)
SmSnRK2.3-GST-R	GACGTCGACTCACATTGCATAAATG (*Sal*I site underlined)
SmSnRK2.6-GST-F	CGGGATCCATGGATCGACC (*BamH*I site underlined)
SmSnRK2.6-GST-R	GACGTCGACTCACATAGCATAGAC (*Sal*I site underlined)

Table S7. Primers used to identify transgenic lines overexpressing *SmSnRK2.3*, *SmSnRK2.6* and *SmAREB1*

Primer	Sequence(5'-3')	SmSnRK2.3-OEs	SmSnRK2.6-OEs	SmAREB1-OEs
CaMV35S-F	AGATGCCTCTGCCGACAGTGGT	+	+	+
SmSnRK2.3-OE-R	GGACTAGTTCACATTGCATAAATGAC	+		
SmSnRK2.6-OE-R	GGACTAGTTCACATAGCATAGAC		+	
SmAREB1-OE-R	GGACTAGTCTACCAAGGTCCGGTAAG			+
rolB F	GCTCTTGCAGTGCTAGATTT	+	+	+
rolB R	GAAGGTGCAAGCTACCTCTC	+	+	+
rolC F	CTCCTGACATCAAACTCGTC	+	+	+
rolC R	TGCTTCGAGTTATGGGTACA	+	+	+
hptII-F	CGCTTCTGCGGGCGATTTGTG	+	+	+
hptII-R	GCTCTCGGAGGGCGAAGAATC	+	+	+

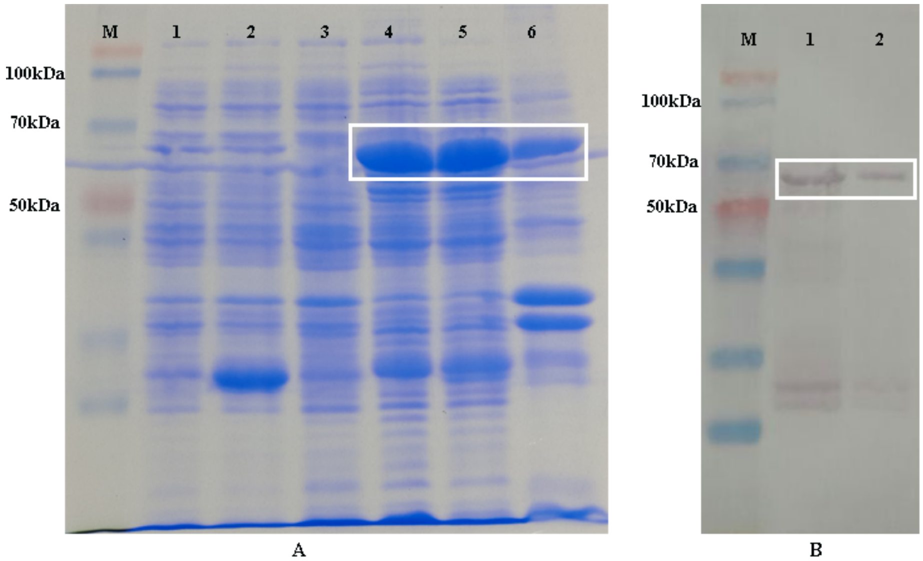

Figure S1 | The detection of SmSnRK2.3 protein expression using SDS-PAGE and western blot. (A) Analysis of SmSnRK2.3 fusion protein expression via SDS-PAGE. Lane M: protein marker; lane 1: bacterial proteins from the *E. coli* Rosetta cells harboring empty pGEX-6p-1 vector uninduced with IPTG; lane 2: bacterial proteins from the *E. coli* Rosetta cells harboring empty pGEX-6p-1 vector induced with 0.5 mM IPTG; lane 3: bacterial proteins from the *E. coli* Rosetta cells harboring the constructed vector SmSnRK2.3-GST uninduced with IPTG; lane 4: bacterial proteins from the *E. coli* Rosetta cells harboring the constructed vector SmSnRK2.3-GST induced with 0.5 mM IPTG; lane 5: fusion protein SmSnRK2.3-GST in supernatant after ultrasonic disruption; lane 6: fusion protein SmSnRK2.3-GST in lower sediment after ultrasonic disruption. The targeted proteins were boxed. **(B)** The purified SmSnRK2.3 protein was verified via western blotting. M: protein marker. The targeted proteins were boxed.

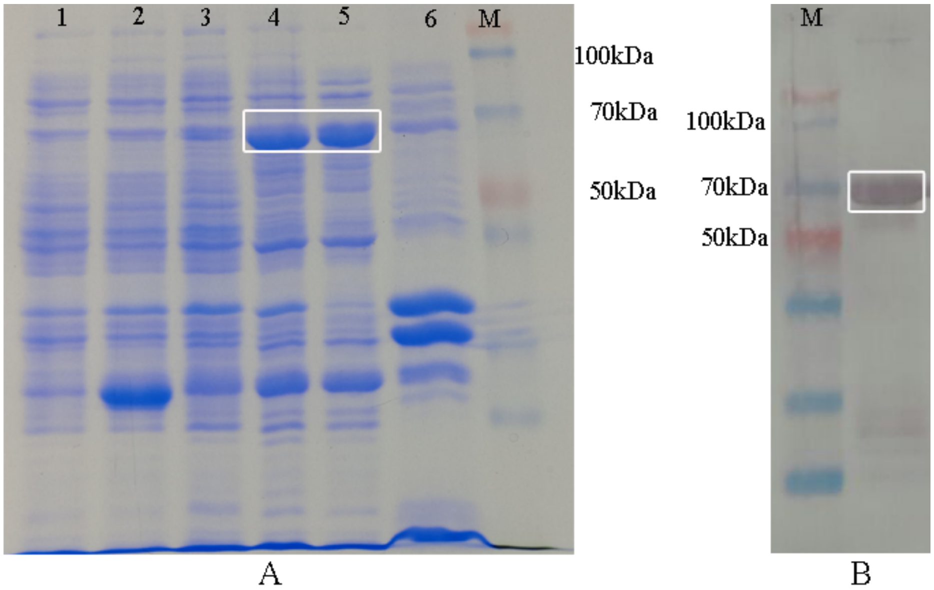

Figure S2 | The detection of SmSnRK2.6 protein expression using SDS-PAGE and western blot. (A) Analysis of SmSnRK2.6 fusion protein expression via SDS-PAGE. Lane M: protein marker; lane 1: bacterial proteins from the *E. coli* Rosetta cells harboring empty pGEX-6p-1 vector uninduced with IPTG; lane 2: bacterial proteins from the *E. coli* Rosetta cells harboring empty pGEX-6p-1 vector induced with 0.5 mM IPTG; lane 3: bacterial proteins from the *E. coli* Rosetta cells harboring the constructed vector SmSnRK2.6-GST uninduced with IPTG; lane 4: bacterial proteins from the *E. coli* Rosetta cells harboring the constructed vector SmSnRK2.6-GST induced with 0.5 mM IPTG; lane 5: fusion protein SmSnRK2.6-GST in supernatant after ultrasonic disruption; lane 6: fusion protein SmSnRK2.6-GST in lower sediment after ultrasonic disruption. The targeted proteins were boxed. **(B)** The purified SmSnRK2.6 protein was verified via western blotting. M: protein marker. The targeted protein was boxed.
